# Efficacy of intratympanic methylprednisolone versus standard therapy in adult sudden sensorineural hearing loss

**DOI:** 10.3389/fneur.2025.1743157

**Published:** 2025-12-19

**Authors:** Xiang-Da Meng, Ting-Ting Li, Bu-Tian Zhang

**Affiliations:** 1 Department of Otolaryngology, The First Affiliated Hospital of Qiqihar Medical College, Qiqihar, Heilongjiang, China; 2 Department of Otolaryngology-Head and Neck Surgery, The Fourth Affiliated Hospital of Harbin Medical University, Harbin, Heilongjiang, China

**Keywords:** hearing recovery, intratympanic methylprednisolone, quality of life, standard treatment, sudden sensorineural hearing loss

## Abstract

**Background:**

Sudden sensorineural hearing loss (SSNHL) is a medical emergency that can significantly impact quality of life. The study aimed to compare the efficacy and safety of intratympanic methylprednisolone therapy (IMT) with standard treatment (ST) in adult patients suffering from unilateral SSNHL.

**Methods:**

A retrospective analysis was conducted on 300 adult patients diagnosed with unilateral SSNHL, treated at our hospital from June 2022 to November 2024. Patients were divided into two groups based on their treatment protocols: IMT group (142 patients) and ST group (158 patients). All patients completed 1 year of follow-up. Outcomes were assessed via pure tone average (PTA), word recognition score (WRS), tinnitus/dizziness resolution, and quality of life (SF-36).

**Results:**

The IMT group showed significantly greater improvement in PTA and WRS at both follow-up points compared to the ST group (*p* < 0.05). Tinnitus reduction was also significantly better in the IMT group at 10 days and 8 weeks (*p* < 0.01). Overall treatment efficacy (cured + markedly effective + effective) was significantly higher with IMT (*p* = 0.031), especially in severe cases (*p* = 0.034). ST caused more systemic side effects like gastrointestinal issues and blood sugar problems (*p* < 0.05). IMT caused more local ear discomfort (*p* < 0.001). Quality of life (SF-36) scores for physical functioning, role-physical, and social functioning were significantly better in the IMT group (*p* < 0.05). Logistic regression confirmed IMT significantly reduced the risk of ineffective treatment.

**Conclusion:**

IMT demonstrated superior efficacy and an acceptable safety profile compared to ST for adult patients with unilateral SSNHL, suggesting it as a preferable therapeutic option.

## 1 Introduction

Sudden sensorineural hearing loss (SSNHL) is a frightening condition which hearing drops quickly, usually in one ear, often without a clear cause. It’s defined as losing at least 30 decibels (dB) of hearing over three connected sound frequencies within 3 days (1, 2). This affects roughly 5 to 20 people per 100,000 each year. Beyond the hearing loss itself, SSNHL frequently causes ringing in the ears (tinnitus), dizziness, and a feeling of fullness in the ear. These problems can really impact a person’s ability to communicate, work, and enjoy life, leading to stress and reduced quality of life (3, 4).

For many years, the main treatment for SSNHL has been corticosteroids taken by mouth or through a vein (systemic steroids). These drugs, like prednisone, work by reducing inflammation and swelling, which are thought to be key parts of what causes SSNHL. Often, doctors also add other medicines like drugs to improve blood flow (e.g., ginkgo biloba extract) or nerve function (e.g., gastrodin). This combination is often called standard therapy (ST) (5).

However, systemic steroid treatment has significant downsides. Taking high doses of steroids throughout the body can cause unpleasant side effects like stomach upset, increased blood sugar levels (especially risky for people with diabetes), trouble sleeping, and mood changes. For some patients with health problems like uncontrolled diabetes or severe high blood pressure, these side effects might even make systemic steroids unsafe to use (6, 7).

To avoid these whole-body side effects, doctors have developed a different way to deliver steroids: directly into the middle ear space. This is called intratympanic steroid therapy (IST). By injecting the steroid (like methylprednisolone) through the eardrum, the drug can reach the inner ear fluid (perilymph) at much higher concentrations than what’s possible with pills or IVs. The idea is that this local delivery targets the problem area more directly while minimizing exposure to the rest of the body (8, 9).

While IST seems promising, there’s still debate about how well it works compared to the standard systemic approach, especially over time. Studies have had mixed results. We also need more information about its effects on related symptoms like tinnitus and overall quality of life, and how it performs in patients with more severe hearing loss (10).

Therefore, this study aimed to compare the effectiveness and safety of intratympanic methylprednisolone (IMT) directly against the standard systemic therapy (ST) in adults experiencing SSNHL. We looked closely at hearing recovery, improvement in tinnitus and dizziness, side effects, and how patients felt about their daily lives after treatment. The findings could reshape treatment paradigms, particularly for patient intolerant to systemic steroids or with severe SSNHL.

## 2 Materials and methods

### 2.1 Case selection

This study conducted a retrospective analysis of 300 adult patients with unilateral sudden sensorineural hearing loss (SSNHL) who were treated at our hospital from June 2022 to November 2024. Patients were divided into two groups based on their treatment protocols: those who received intratympanic methylprednisolone therapy (IMT) were defined as the IMT group, comprising 142 patients, while those who received standard treatment (ST) were defined as the ST group, comprising 158 patients. All patients completed 1 year of follow-up.

This study was approved by the Institutional Review Board and Ethics Committee of our institution. Given that the data used in this study consisted of anonymized patient information and posed no potential risks or impacts on the participants, it was determined that individual informed consent was not required. This exemption was based on relevant regulations and ethical guidelines for retrospective studies and was jointly determined by our Institutional Review Board and Ethics Committee. These measures ensured that the study complied with both regulatory requirements and ethical standards, providing robust and ethically sound data for analysis.

### 2.2 Selection criteria

#### 2.2.1 Inclusion criteria

(1) Patients diagnosed with unilateral sudden sensorineural hearing loss (SSNHL), as confirmed by pure-tone audiometry showing a hearing loss of ≥30 dB across three consecutive frequencies, and adhering to the established criteria for diagnosing and classifying SSNHL following thorough otolaryngological examination (5); (2) Symptoms appearing no more than 7 days prior to presentation; (3) Absence of any treatment involving neurotrophic drugs, corticosteroids, anti-infective agents, anticoagulants, or immunomodulator drugs in the preceding 4 weeks; (4) Patients aged from 18 to 65 years; and (5) Comprehensive medical records and follow-up data.

#### 2.2.2 Exclusion criteria

Patients were excluded if they had any of the following conditions: (1) Structural abnormalities of the outer, middle, or inner ear; dysfunction of the eustachian tube; or trauma or surgical history affecting the affected ear; (2) Previous instances of auditory impairment, including age-associated or congenital hearing decline; (3) The primary diseases involve the hematologic system or digestive system; (4) Significant hypertension or diabetes not adequately controlled, which contraindicates the administration of corticosteroid pulse treatment; and (5) Women who are pregnant or breastfeeding, or individuals diagnosed with a psychiatric disorder.

### 2.3 Data extraction

Data were extracted from the electronic medical record (EMR) system and included patient demographic characteristics (such as age, gender, and ethnicity), baseline health status (including the presence of tinnitus, vertigo, and baseline hearing assessment results), treatment-related information, follow-up and recovery status. All data were recorded using standardized data collection forms and verified by two individuals to ensure accuracy.

### 2.4 Intratympanic methylprednisolone therapy protocol

Patients in the intratympanic methylprednisolone therapy (IMT) group received injections of methylprednisolone (Pfizer, United States, National Drug Approval Number: H20190405, 40 mg). The procedure was performed with the patient lying supine, ensuring that the ear in question was oriented upwards. Following removing earwax, 70% alcohol was used to sanitize the external auditory canal. Topical anesthesia of the tympanic membrane was achieved using 2% lidocaine for 3 to 5 min.

Before use, heated the injection solution to an appropriate temperature. With the aid of an endoscopic, a myringotomy was executed in the anterior-inferior portion of the tympanic membrane, and then 1 mL (20 mg) of methylprednisolone was injected into the middle ear. Following the injection, patients were instructed to remain in a supine position for 30 min to 1 h, avoiding swallowing and speaking to keep the medication remained in the middle ear for the longest possible duration. The injections were administered every alternate day, totaling five injections. An alcohol-soaked cotton ball was placed in the external auditory canal after each injection and removed the next day. Throughout the treatment phase, patients were instructed to maintain the external auditory canal in a dry state and to prevent colds or upper respiratory tract infections. These precautions ensured optimal conditions for drug retention and effectiveness, minimizing potential complications associated with the treatment.

### 2.5 Standard treatment protocol

Patients underwent the following systemic treatments: Oral administration of prednisone (60 mg, produced by Shandong Xinhua Pharmaceutical Co., Ltd., National Drug Approval Number: H37020647, 5 mg per tablet) once daily in the morning, with a dosage reduction of 10 mg every 3 days. Additionally, patients received an intravenous infusion of ginkgo biloba extract (87.5 mg, produced by Taiwan Chisun Chemical & Pharmaceutical Co., Ltd., National Drug Approval Number: HC20181022, 5 mL containing 17.5 mg), administered once daily. Intravenous infusion of 0.6 g gastrodin (Dandong Yichuang Pharmaceutical Co., Ltd., National Drug Approval Number: H20013046, 2 mL:0.2 g), once daily. It should be noted that while ginkgo biloba extract and gastrodin are not universally recognized as standard therapy in international guidelines, they are commonly used as adjunctive treatments in Chinese clinical practice for SSNHL, based on national guidelines and local consensus regarding their potential vasoactive and neuroprotective effects (11, 12). All the above medications were diluted in 250 mL of normal saline and administered via intravenous drip for 10 consecutive days.

### 2.6 Follow-up strategy

Upon completing a 10-day systemic treatment course, patients were scheduled for follow-up audiometry. If their hearing had fully recovered, no additional treatment was necessary. For those who did not achieve complete hearing restoration, prescriptions for oral medications, specifically ginkgo biloba extract (7.5 mg) and mecobalamin tablets (0.5 mg) were provided, to be taken three times daily over 4 weeks. Audiometric tests were repeated at the four-week mark. If full recovery was confirmed, medication was stopped. Patients still experiencing hearing deficits continued treatment for an additional 8 weeks. After this period, all medications were discontinued, and a final hearing assessment was conducted at the eight-week follow-up. Hearing status at 8 weeks post-discharge served as the ultimate indicator of treatment success. Throughout the follow-up phase, besides audiometric tests, otolaryngological examinations were also carried out to check for potential complications, including tympanic membrane perforation or secondary acute or chronic otitis media. Our follow-up assessments were conducted at 10 days and 8 weeks post-treatment, time points commonly used in prior studies to capture early and intermediate recovery (13, 14).

### 2.7 Outcome measures and efficacy criteria

(1) Auditory assessments using Pure Tone Average (PTA) were conducted at three time points: before treatment initiation, 10 days and 8 weeks post-treatment. Air conduction thresholds at frequencies of 0.5, 1, 2, and 4 kHz were measured in triplicate, and the average value was computed to determine the PTA (15). Word Recognition Score (WRS) was assessed at the same time points (baseline, 10 days, and 8 weeks) using monosyllabic word lists presented via monitored live voice or recorded speech at 40 dB sensation level (SL) above the patient’s speech reception threshold (SRT) (16). Patients were instructed to repeat each word, and the percentage of correctly identified words was recorded as the WRS. Testing was conducted in a sound-treated booth to minimize ambient noise interference. The severity of hearing loss was categorized according to the average thresholds at 500 Hz, 1,000 Hz, 2000 Hz, and 4,000 Hz (17): Mild: 20–34 dB HL, Moderate: 35–49 dB HL, Moderate to severe: 50–64 dB HL, Severe: 65–79 dB HL, Profound: 80–94 dB HL, and Total deafness: ≥95 dB HL.
(2) Hearing outcomes were analyzed for all patients, including PTA values at baseline, 10 days post-treatment, and at 8 weeks. In addition to PTA, hearing thresholds for affected frequencies were evaluated. For patients experiencing total frequency hearing loss or complete deafness, the mean threshold across tested frequencies was determined. In cases of low- and high-frequency hearing loss, only the mean threshold for the affected frequencies was calculated. If no response was detected at the audiometer’s maximum output, both PTA and the mean threshold for the affected frequencies were recorded as 120 dB HL.

Hearing loss types:

Low-frequency hearing loss: Characterized by hearing impairment at frequencies up to 1 kHz, with a minimum loss of 20 dB HL at 0.25 and 0.5 kHz. The average air conduction thresholds at 0.25–0.5 kHz are at least 20 dB higher than those at 4–8 kHz.
High-frequency hearing loss: Defined by hearing impairment at frequencies starting from 2 kHz, with a minimum loss of 20 dB HL at 4 and 8 kHz. The average air conduction thresholds at 4–8 kHz are at least 20 dB higher than those at 0.25–0.5 kHz.
Flat loss: Uniform hearing loss across all frequencies, with an average threshold below 80 dB HL (measured at 250, 500, 1,000, 2000, 3,000, 4,000, and 8,000 Hz).
Total deafness: Consistent hearing loss across all frequencies, with an average threshold of 81 dB HL or higher (measured from 250 to 8,000 Hz).

(3) The efficacy of the treatment was evaluated based on the recovery of hearing thresholds at the affected frequencies:

 Cured: Hearing thresholds returned to normal levels or matched those of the contralateral healthy ear.
 Markedly effective: Improvement in hearing exceeded 30 dB HL.
 Effective: Improvement in hearing ranged from 15 to 30 dB HL.
 Ineffective: Improvement in hearing was less than 15 dB HL.

The overall efficacy rate was calculated as the proportion of patients classified as cured, markedly effective, or effective relative to the total number of patients:

Efficacy rate = (number of patients cured + number of patients markedly effective + number of patients effective)/Total number of patients×100%.

### 2.8 Assessment of quality of life

To evaluate the quality of life, the SF-36 questionnaire was utilized. This instrument assesses eight domains: Physical Functioning (PF), Role Limitations due to Physical Health (RP), Bodily Pain (BP), General Health Perceptions (GH), Vitality (VT), Social Functioning (SF), Role Limitations due to Emotional Problems (RE), and Mental Health (MH). Scores for each domain range from 0 to 100, with higher scores reflecting better quality of life. The reliability confirmed by Cronbach’s alpha coefficients exceeded 0.70 (18).

### 2.9 Statistical analysis

Data analysis was performed using SPSS 29.0 statistical software (SPSS Inc., Chicago, IL, United States). Categorical data are reported as [*n* (%)]. Chi-square tests using the basic formula, with the test statistic denoted as χ^2^, were conducted when the sample size was ≥40 and the theoretical frequency T was ≥5. When the sample size was ≥40 but the theoretical frequency was 1 ≤ T < 5, a corrected formula was used to adjust the chi-square test. For sample sizes smaller than 40 or when the expected frequency (T) was less than 1, Fisher’s exact test was employed for statistical analysis. Continuous variables were initially tested for normal distribution using the Shapiro–Wilk test. Normally distributed continuous data are presented in the format of (X ± s). Comparisons of continuous variables between two groups were performed using unpaired *t*-tests for normally distributed data. A multifactorial logistic regression analysis was performed to identify factors affecting Ineffective Treatment Outcome. A *p* value <0.05 was considered statistically significant.

## 3 Results

### 3.1 Basic data

Table 1 presents a comparison of demographic characteristics between the ST Group (*n* = 158) and the IMT Group (*n* = 142). The parameters analyzed include gender distribution, age, body mass index (BMI), ethnicity, educational level, residence, smoking history, drinking history, neurological symptoms, cardiovascular disease, and metabolic disease. The results indicate no statistically significant differences between the two groups in any of the parameters assessed, as shown by *p* values greater than 0.05 for all comparisons. These findings suggest that the baseline demographic characteristics and health profiles of participants in the ST and IMT Groups are similar, indicating a balanced distribution of these factors which could help minimize confounding variables in subsequent analyses.

**Table 1:** Comparison of demographic characteristics between two groups.

Parameters	ST group (*n* = 158)	IMT group (*n* = 142)	t/χ2	*P*
Male/Female [*n* (%)]	85 (53.80%)	78 (54.93%)	0.039	0.844
Age (years)	44.87 ± 9.15	45.26 ± 9.02	0.378	0.706
BMI (kg/m^2^)	21.82 ± 3.12	21.35 ± 3.08	1.307	0.192
Ethnicity (Han/Other) [*n* (%)]	132 (83.54%)	120 (84.51%)	0.052	0.820
Educational level (high school or below/junior college or above) [*n* (%)]	105 (66.46%)	92 (64.79%)	0.092	0.761
Residence (Urban/Rural)	103 (65.19%)	94 (66.2%)	0.034	0.854
Smoking history [*n* (%)]	35 (22.15%)	33 (23.24%)	0.050	0.822
Drinking history [*n* (%)]	19 (12.03%)	19 (13.38%)	0.124	0.725
Neurological symptoms [*n* (%)]	18 (11.39%)	14 (9.86%)	0.185	0.668
Cardiovascular disease [*n* (%)]	73 (46.20%)	64 (45.07%)	0.039	0.844
Metabolic disease [*n* (%)]	16 (10.13%)	13 (9.15%)	0.081	0.776

BMI, body mass index.

The clinical characteristics of the ST group and the IMT group were compared (Table 2). There was no significant difference in the distribution of affected side (left/right) between the two groups, with similar proportions observed (*p* = 0.846). The duration of disease was also comparable (*p* = 0.657). Regarding the type of hearing loss, no statistically significant difference was found (*p* = 0.976), with similar distributions observed for low-frequency loss, high-frequency loss, flat loss, and total deafness in both groups. These results indicate that there are no significant differences in clinical characteristics between the ST and IMT groups.

**Table 2:** Comparison of clinical characteristics between two groups.

Parameters	ST group (*n* = 158)	IMT group (*n* = 142)	*t*/χ2	*P*
Side (left/right) [*n* (%)]	75 (47.47%)	69 (48.59%)	0.038	0.846
Duration of disease (days)	4.20 ± 1.05	4.25 ± 1.15	0.445	0.657
Type of hearing loss			0.210	0.976
Low-frequency loss [*n* (%)]	37 (23.42%)	35 (24.65%)		
High-frequency loss [*n* (%)]	30 (18.98%)	25 (17.61%)		
Flat loss [*n* (%)]	64 (40.51%)	56 (39.44%)		
Total deafness [*n* (%)]	27 (17.09%)	26 (18.30%)		

### 3.2 Treatment outcomes

#### 3.2.1 PTA changes

**Figure 1:**
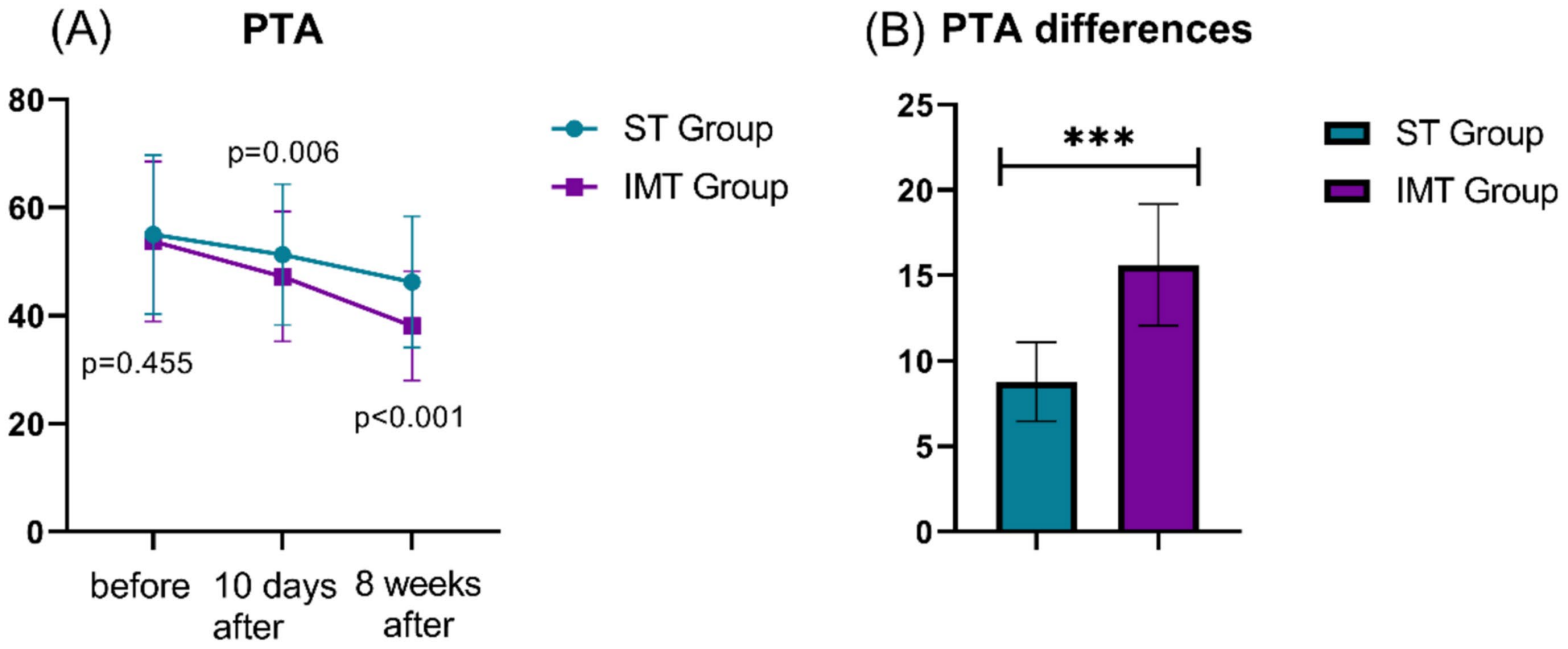
Comparison of pre- and post-treatment PTA differences between two groups. **(A)**: PTA; **(B)**: PTA differences; PTA, pure tone average; ***: *p* < 0.001.

Before treatment, the PTA values were not significantly different between the two groups (*p* = 0.455) (Figure 1). However, significant differences were observed at both the 10-day and 8-week post-treatment evaluations. At 10 days post-treatment, the IMT group demonstrated greater improvement compared to the ST group, with a statistically significant difference (t = 2.795, *p* = 0.006). This disparity became more pronounced at 8 weeks post-treatment, where the IMT group achieved significantly lower PTA values than the ST group, showing a highly significant difference (t = 6.306, *p* < 0.001). The overall PTA improvement, measured as the difference between pre- and post-treatment values, was also significantly greater in the IMT group compared to the ST group, with a notably high significance (*t* = 19.477, *p* < 0.001). These findings indicate that both interventions improved hearing thresholds, but the IMT protocol produced substantially greater and more sustained therapeutic effects over time. This indicates that IMT could be more effective than ST in the treatment of hearing loss.

#### 3.2.2 WRS changes

**Figure 2:**
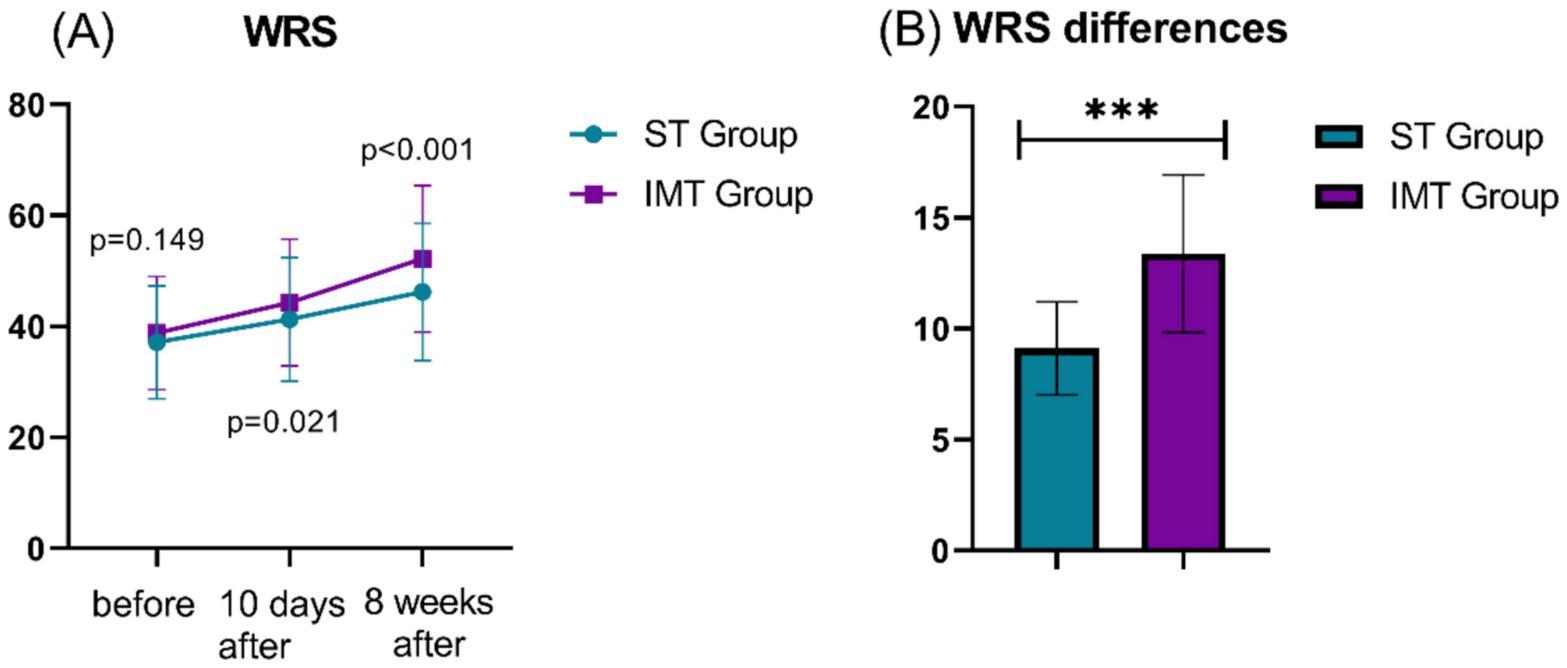
Comparison of pre- and post-treatment WRS differences between two groups. **(A)**: WRS; **(B)**: WRS differences; WRS, word recognition score; ***: *p* < 0.001.

At baseline, no significant difference was observed in WRS scores between the two groups (*p* = 0.149) (Figure 2). By 10 days post-treatment, both groups demonstrated improvement, but the IMT group showed superior gains (t = 2.326, *p* = 0.021). At 8 weeks post-treatment, the IMT group achieved significantly higher WRS scores compared to the ST group, with the largest between-group difference (t = 4.059, *p* < 0.001). Analysis of WRS differences (pre-to-post treatment changes) confirmed a markedly greater improvement in the IMT group than the ST group, reaching the highest statistical significance (t = 12.474, *p* < 0.001). These results indicate that while both interventions enhanced speech recognition abilities, the IMT protocol yielded faster and more substantial improvements, particularly over the long term.

#### 3.2.3 Tinnitus and dizziness conditions

Initially, there was no significant difference in tinnitus prevalence between the two groups pre-treatment (*p* = 0.844) (Table 3). However, post-treatment assessments revealed a different outcome. At 10 days after treatment, a significant difference emerged, favoring the IMT group (χ^2^ = 9.265, *p* = 0.002). This trend became even more pronounced at 8 weeks post-treatment, with an even greater distinction observed between the groups (χ^2^ = 17.435, *p* < 0.001). These findings indicate that while both treatments contributed to reducing tinnitus, the IMT approach was significantly more effective over time.

**Table 3:** Comparison of the frequency of tinnitus between two groups.

Parameters	ST group (*n* = 158)	IMT group (*n* = 142)	*t*	*P*
Pre-treatment	104 (65.82%)	95 (66.90%)	0.039	0.844
10 days post-treatment	48 (30.38%)	22 (15.49%)	9.265	0.002
8 weeks post-treatment	28 (17.72%)	4 (2.82%)	17.435	<0.001

Prior to treatment, there was no significant difference in the prevalence of dizziness between the two groups pre-treatment (*p* = 0.798) (Table 4). Post-treatment evaluations at 10 days showed a notable decrease in the incidence of dizziness for both groups, yet the difference remained non-significant (*p* = 0.731). Similarly, by 8 weeks post-treatment, the reduction in dizziness continued without any significant difference emerging between the groups (*p* = 0.837). These findings indicate that both treatments were equally effective in reducing the occurrence of dizziness over time.

**Table 4:** Comparison of the frequency of dizziness between two groups.

Parameters	ST group (*n* = 158)	IMT group (*n* = 142)	*t*	*P*
Pre-treatment	59 (37.34%)	51 (35.92%)	0.066	0.798
10 days post-treatment	8 (5.06%)	6 (4.23%)	0.118	0.731
8 weeks post-treatment	5 (3.16%)	3 (2.11%)	0.042	0.837

### 3.3 Treatment efficacy assessment

For all patients, the overall efficacy rate was 56.96% in the ST group versus 69.01% in the IMT group, with a statistically significant difference indicated by χ^2^ = 4.643 and *p* = 0.031 (Table 5). The breakdown of outcomes shows that the IMT group had higher counts in the “Cure” and “Markedly Effective” categories compared to the ST group, while the ST group showed slightly higher numbers in the “Effective” and “Ineffective” categories.

**Table 5:** Comparison of efficacy between two groups.

Category	Treatment	Cure	Markedly effective	Effective	Ineffective	Efficacy rate (%)	χ2	*P*
All	ST (*n* = 158)	14	48	28	67	56.96%	4.643	0.031
	IMT (*n* = 142)	29	53	16	46	69.01%		
Severe and above	ST (*n* = 91)	9	14	13	54	39.56%	4.503	0.034
	IMT (*n* = 82)	19	17	12	36	58.54%		

Severe and above patients refer to those with Flat loss and Total deafness.

When focusing on patients with severe conditions and above, similar trends were observed. The efficacy rate for the ST group was 39.56%, significantly lower than the 58.54% observed in the IMT group (χ^2^ = 4.503, *p* = 0.034). Specifically, the IMT group demonstrated superior outcomes in terms of both “Cure” and “Markedly Effective” rates compared to the ST group, whereas the ST group reported higher rates of “Ineffective” cases. These results highlight that the IMT treatment not only achieves a higher overall efficacy rate but also performs better in managing more severe cases compared to the ST treatment.

### 3.4 Safety assessment

**Figure 3:**
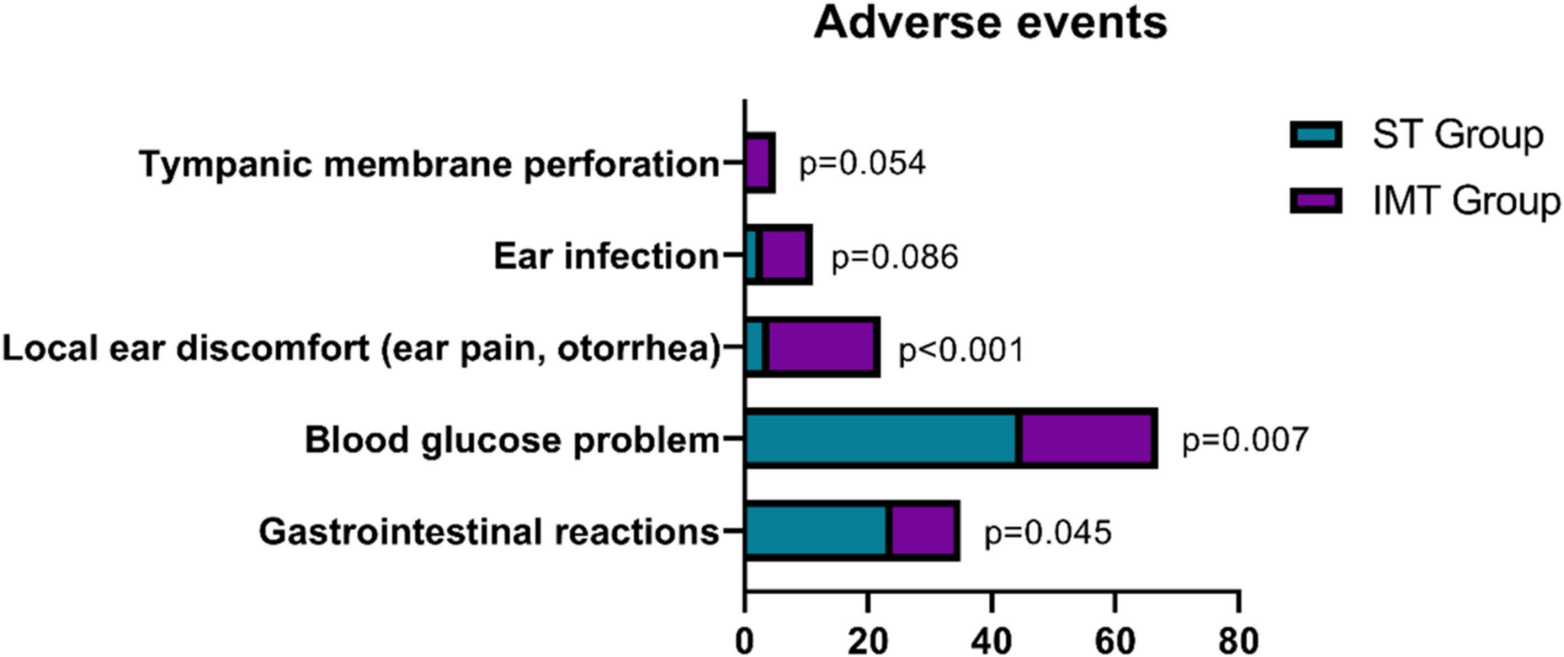
Comparison of adverse events between two groups.

For gastrointestinal reactions, there was a significant difference between the groups (χ^2^ = 4.021, *p* = 0.045), with a higher incidence in the ST group compared to the IMT group (Figure 3). Blood glucose problems also showed a significant difference (χ^2^ = 7.273, *p* = 0.007), again with more cases reported in the ST group. Local ear discomfort, including symptoms such as ear pain and otorrhea, was significantly more frequent in the IMT group (χ^2^ = 11.325, *p* < 0.001). In contrast, ear infections were slightly more common in the IMT group, but this difference did not reach statistical significance (*p* = 0.086). Lastly, tympanic membrane perforation occurred exclusively in the IMT group, though this finding approached but did not meet statistical significance (*p* = 0.054). Despite the higher frequency of local ear-related adverse events in the IMT group, these findings suggest that IMT is associated with fewer severe systemic adverse events compared to ST. These results underscore the potential benefits of IMT, suggesting it as a preferable therapeutic choice when balancing efficacy and safety profiles.

### 3.5 Quality of life

For PF, the IMT group scored significantly higher than the ST group (t = 3.276, *p* = 0.001) (Table 6). Similarly, RP scores were also significantly better in the IMT group (t = 2.647, *p* = 0.009), indicating that patients treated with IMT experienced fewer limitations due to physical health problems. In contrast, no significant differences were observed for BP, GH, VT, or MH (all *p* > 0.05). However, SF scores were significantly higher in the IMT group compared to the ST group (t = 2.497, *p* = 0.013), suggesting better social well-being among IMT recipients. No significant difference was found in RE scores (*p* = 0.766). Overall, these findings demonstrate that the IMT treatment not only leads to better clinical outcomes but also enhances multiple aspects of patients’ quality of life more effectively than the ST treatment.

**Table 6:** Comparison of SF-36 score between two groups.

Parameters	ST group (*n* = 158)	IMT group (*n* = 142)	*t*	*P*
PF	67.22 ± 15.61	72.52 ± 12.31	3.276	0.001
RP	59.44 ± 17.23	64.23 ± 14.12	2.647	0.009
BP	74.26 ± 15.33	74.82 ± 15.26	0.318	0.751
GH	67.54 ± 14.92	68.31 ± 13.72	0.462	0.645
VT	69.81 ± 13.52	70.62 ± 11.91	0.549	0.583
SF	63.21 ± 16.34	67.48 ± 13.21	2.497	0.013
RE	64.48 ± 18.11	65.05 ± 15.02	0.297	0.766
MH	73.91 ± 12.46	73.57 ± 11.92	0.241	0.810

SF-36: the short form 36 health survey questionnaire; PF: Physical Functioning; RP: Role-Physical; BP: Bodily Pain; GH: General Health; VT: Vitality; SF: Social Functioning; RE: Role-Emotional; MH: Mental Health.

### 3.6 Multifactorial logistic regression analysis of factors affecting ineffective treatment outcome

A multifactorial logistic regression analysis was conducted to identify factors associated with ineffective treatment outcomes (Table 7). The use of IMT was significantly associated with a reduced risk of ineffective treatment compared to ST (*p* < 0.001). Baseline PTA also emerged as a significant predictor, where each 1-dB increase in baseline PTA was linked to a higher likelihood of ineffective treatment (*p* < 0.001). Similarly, baseline WRS had a significant inverse relationship with ineffective treatment outcomes (*p* < 0.001). For each 1% increase in baseline WRS, the odds of ineffective treatment decreased, indicating that better initial word recognition ability is associated with more favorable treatment results. Age showed a trend toward significance but did not reach statistical significance (*p* = 0.105). BMI and duration of disease did not show significant associations with treatment ineffectiveness (*p* > 0.05). In summary, this analysis highlights that treatment type (IMT vs. ST), baseline PTA, and baseline WRS are significant predictors of treatment effectiveness.

**Table 7:** Multifactorial logistic regression analysis of factors affecting ineffective treatment outcome.

Variable	Coefficient	Std error	Wald stat	*P*	OR (95%CI)
Treatment type (IMT vs. ST)	−0.824	0.250	10.75	<0.001	0.439 (0.273–0.705)
Age (per 1-year increase)	0.018	0.011	2.63	0.105	1.018 (1.000–1.036)
BMI (per 1-kg/m^2^ increase)	0.130	0.240	0.29	0.590	1.139 (0.694–1.869)
Baseline PTA (per 1-dB increase)	0.045	0.016	8.12	<0.001	1.046 (1.022–1.071)
Baseline WRS (per 1% increase)	−0.020	0.007	8.160	<0.001	0.980 (0.967–0.994)
Duration of disease (per 1-day increase)	0.280	0.270	1.06	0.303	1.323 (0.874–2.001)

## 4 Discussion

The present study compares the efficacy and safety of intratympanic methylprednisolone therapy (IMT) with standard treatment (ST) in adult patients suffering from unilateral sudden sensorineural hearing loss (SSNHL). The findings suggest that IMT offers a more effective therapeutic option for SSNHL compared to ST, particularly in severe cases. The multifactorial logistic regression analysis confirmed that receiving IMT itself was a strong independent predictor of treatment success, significantly reducing the risk of an ineffective outcome.

The fundamental advantage of IMT lies in its ability to overcome the blood-labyrinth barrier (BLB). The BLB tightly regulates the passage of substances from the systemic circulation into the inner ear fluids (perilymph). Systemic corticosteroids, administered orally or intravenously as in the ST group, face significant difficulty crossing this barrier (19). Consequently, only a small fraction (typically <1–2%) of the high systemic dose actually reaches the perilymph, resulting in suboptimal drug concentrations at the primary site of injury in SSNHL – the cochlea. In contrast, IMT delivers methylprednisolone directly into the middle ear space. From here, the drug diffuses across the round window membrane, achieving perilymph concentrations estimated to be 100 to 1,000 times higher than those attainable via systemic administration. This massive local concentration gradient is pharmacologically crucial (20, 21).

SSNHL is widely believed to involve inflammatory processes, oxidative stress, microvascular compromise, and potential viral insults within the cochlea. Methylprednisolone, a potent glucocorticoid, exerts its therapeutic effects through multiple mechanisms relevant to these pathologies. Firstly, it acts as a powerful anti-inflammatory agent, rapidly suppressing the production and release of pro-inflammatory cytokines (such as TNF-*α* and IL-1β) and inhibiting the infiltration of immune cells into cochlear tissues. This reduces inflammation-mediated damage to delicate hair cells, supporting cells, and the stria vascularis. Secondly, methylprednisolone possesses significant antioxidant properties, neutralizing damaging free radicals generated during ischemic or inflammatory events that contribute to cellular injury and apoptosis (19, 22). Thirdly, it may help stabilize cochlear blood flow and endothelial function, counteracting potential microvascular ischemia. Finally, it stabilizes neural membranes. The exceptionally high perilymph concentrations achieved with IMT maximize these protective mechanisms during the critical early phase of SSNHL, when hair cells and neurons may still be salvageable. This direct and potent action at the target organ explains the significantly faster and greater improvements in both PTA (reflecting cochlear sensitivity) and WRS (reflecting auditory processing and speech understanding) observed in the IMT group. The pronounced benefit in severe cases underscores the importance of delivering high-dose therapy directly to the cochlea when damage is extensive (23, 24).

The significantly superior reduction of tinnitus in the IMT group is another critical finding. Tinnitus in SSNHL often arises from aberrant neural activity generated or triggered by the initial cochlear damage. By more effectively suppressing the underlying cochlear inflammation, oxidative stress, and neural excitotoxicity at the peripheral source, IMT likely reduces this aberrant neural signaling and prevents maladaptive central plasticity more effectively than systemic therapy (25, 26). The sustained advantage over ST at both 10 days and 8 weeks suggests that early, high-concentration local treatment has a more profound and lasting impact on the pathological processes generating tinnitus. In contrast, the comparable resolution of dizziness between groups suggests that vestibular compensation mechanisms might function effectively regardless of the steroid delivery route, or that the vestibular system responds similarly to the steroid levels achieved by both methods (27–29).

In comparison with other studies, the IMT protocol used in the present study (5 injections of 20 mg methylprednisolone each, administered every other day) aligns with or exceeds the dosages reported in prior research. For instance, studies utilizing dexamethasone often employ doses ranging from 4 to 24 mg per injection, with varying injection frequencies (22). Some studies and meta-analyses have shown that higher steroid concentrations in the perilymph, as achieved with methylprednisolone, are associated with better hearing recovery, especially in severe SSNHL cases (8, 30). Moreover, our findings of superior tinnitus reduction with IMT are consistent with several randomized trials reporting significant symptomatic relief with intratympanic steroids compared to systemic administration (31, 32). These comparisons reinforce the clinical relevance of our protocol and support its efficacy in both auditory and symptomatic outcomes.

The safety profile further strengthens the case for IMT. While transient local adverse effects like ear discomfort and minor otorrhea were more frequent in the IMT group, the more serious and potentially debilitating systemic side effects – gastrointestinal disturbances and blood glucose dysregulation – occurred significantly more often in the ST group (33). This difference is physiologically consistent. IMT minimizes systemic exposure to corticosteroids, thereby largely avoiding their well-documented widespread metabolic, immunological, and gastric effects (34, 35). This is a particularly important advantage for patients with comorbidities like diabetes or peptic ulcer disease, who are often excluded from or face significant risks with high-dose systemic steroid therapy. The observed rates of persistent tympanic membrane perforation and otitis media in the IMT group were low and generally manageable, suggesting these are acceptable risks in the context of the significant therapeutic benefits (36).

The enhanced quality of life observed in the IMT group, specifically in Physical Functioning, Role-Physical limitations, and Social Functioning domains of the SF-36, likely reflects the combined impact of better hearing recovery, reduced tinnitus burden, and the avoidance of debilitating systemic side effects. Improved auditory communication facilitates social interaction and participation in daily activities, while feeling physically better naturally enhances perceived physical function and role fulfillment. This finding is particularly important, as SSNHL can profoundly affect daily activities and interpersonal relationships. Enhancing quality of life should be a key objective in managing SSNHL, and IMT seems to offer a promising avenue toward achieving this goal (37).

Several limitations of this study must be acknowledged. The retrospective design inherently carries risks of selection bias and confounding variables. The ST regimen included adjunctive medications (ginkgo biloba extract, gastrodin), making the comparison one between treatment strategies rather than solely isolating the effect of systemic steroids versus local steroids. While this reflects common clinical practice, it complicates attributing differences solely to the steroid component. Future prospective studies directly comparing IMT to systemic steroids alone would be valuable. Additionally, due to the relatively small sample size in certain subgroups, we did not perform a stratified analysis of treatment efficacy based on hearing loss type (low-frequency, high-frequency, flat loss, total deafness). Future studies with larger cohorts are needed to explore whether IMT shows differential benefits across these subtypes. Moreover, the follow-up period was limited to 10 days and 8 weeks post-treatment, which may not capture potential further recovery, especially in the IMT group. Future studies with longer-term assessments (e.g., 12 weeks, 6 months, or 1 year) would provide more comprehensive insights into the sustained efficacy and possible late-phase improvements. Another limitation involves the generalizability of our findings. Our sample consisted solely of Chinese adults treated at a single institution, which limits the applicability of our conclusions to other populations. Therefore, multicenter trials involving diverse populations are necessary to validate our findings and establish universal guidelines for treating SSNHL.

## 5 Conclusion

In conclusion, this study demonstrates that intratympanic methylprednisolone therapy offers superior efficacy and an acceptable safety profile compared to standard treatment for adult patients with unilateral SSNHL. The localized delivery of methylprednisolone appears to enhance hearing recovery, reduce tinnitus, and improve quality of life, making it a preferable therapeutic option, especially for severe cases. However, the potential for local adverse events necessitates cautious patient selection and close monitoring during treatment. Future research should aim to address the limitations identified herein and further elucidate the mechanisms underlying the benefits of IMT, ultimately guiding optimal management strategies for SSNHL.

## Data Availability

The raw data supporting the conclusions of this article will be made available by the authors, without undue reservation.
